# Strong Eu^2+^ light emission in Eu silicate through Eu^3+^ reduction in Eu_2_O_3_/Si multilayer deposited on Si substrates

**DOI:** 10.1186/1556-276X-8-194

**Published:** 2013-04-26

**Authors:** Leliang Li, Jun Zheng, Yuhua Zuo, Buwen Cheng, Qiming Wang

**Affiliations:** 1 State Key Laboratory on Integrated Optoelectronics, Institute of Semiconductors, Chinese Academy of Sciences, Beijing 100083, People's Republic of China

**Keywords:** Eu^2+^ silicate, Multilayer, Photoluminescence, Si-based photonics

## Abstract

Eu_2_O_3_/Si multilayer nanostructured films are deposited on Si substrates by magnetron sputtering. Transmission electron microscopy and X-ray diffraction measurements demonstrate that multicrystalline Eu silicate is homogeneously distributed in the film after high-temperature treatment in N_2_. The Eu^2+^ silicate is formed by the reaction of Eu_2_O_3_ and Si layers, showing an intense and broad room-temperature photoluminescence peak centered at 610 nm. It is found that the Si layer thickness in nanostructures has great influence on Eu ion optical behavior by forming different Eu silicate crystalline phases. These findings open a promising way to prepare efficient Eu^2+^ materials for photonic application.

## Background

Efficient light emission from Si-based structures and devices has drawn worldwide attention with the aim of developing an integrated optoelectronic platform on Si [1-6]. Such light emitters present an attractive application not only for inter-/intrachip optical interconnects but also, e.g., micro-displays and biological detection. Among the different approaches, rare-earth ion-based materials are very promising candidates due to their outstanding optical properties. Recently, it has been demonstrated that erbium silicate has one order of magnitude higher optically active rare-earth ions than those done through doping, without clustering or precipitation [7-10]. This may open new and interesting perspectives for rare-earth applications in photonics.

Among the various rare earths, Eu ions also have been attracting great interest in optoelectronic application because of its intense and stable emission in the visible region. Compared with other trivalent rare-earth ions, Eu^2+^ emission intensity is several orders stronger because of dipole-allowed transition. This makes for the successful application of Eu^2+^ in phosphors [11,12], and electroluminescent devices, by incorporating Eu^2+^ (such as those doped in SiO_2_ and Eu silicate), have been demonstrated [13-15]. Bellocchi et al. have shown that the external quantum efficiency of Eu_2_SiO_4_ can be reached at about 10%, making Eu silicate of great interest for photonic application [16]. However, in their work, Eu silicate was obtained through the complex reaction between the deposited Eu_2_O_3_ film and Si substrate, which would inevitably cause inhomogeneous distribution of Eu silicate.

In this paper, we show that Eu silicate can be fabricated by optimizing the Eu_2_O_3_/Si multilayer nanostructure deposited on Si substrates. Both the structural and optical properties of nanostructures are studied in detail. Through precisely controlling the thickness of Eu_2_O_3_ and Si layer at nanometer scale, the Eu silicate with highly efficient room-temperature (RT) light emission associated to Eu^2+^ ions is obtained after annealing in N_2_ atmosphere.

## Methods

The Eu_2_O_3_ /Si multilayer films with five periods were grown on Si substrates at 400°C by RF magnetron sputtering. The thin films were deposited in 3.0-mTorr Ar atmosphere. The Eu_2_O_3_ layer and Si layer were prepared by alternately sputtering the Eu_2_O_3_ target and Si target. The thickness of Eu_2_O_3_ layers was kept the same in all samples, while the thickness of Si layers was varied in different samples, as shown in Table 1. After deposition, the samples were thermally treated at 1,000°C for 30 s in N_2_ ambient by rapid thermal annealing. Transmission electron microscopy (TEM, Tecnai G2 F20 S-Twin, FEI Company, Hillsboro, OR, USA) was conducted to investigate the samples' morphology. The distribution of elements in the film was detected by scanning TEM (STEM), and crystallization was demonstrated by selected area electron diffraction pattern (SAED). Rutherford backscattering spectrometry (RBS) was carried out to investigate the film composition. The samples' crystalline phases were identified by X-ray diffraction (XRD, D/max 2400, Rigaku Corporation, Tokyo, Japan) measurements. RT photoluminescence (PL) and photoluminescence excitation (PLE) measurements were performed by using a spectrofluorometer (Nano Log, HORIBA Ltd., Minami-Ku, Kyoto, Japan) equipped with a 450-W Xe lamp.

**Table 1:** **Eu**_**2**_**O**_**3**_**/Si multilayer structure**

**Sample**	**Thickness of Eu**_**2**_**O**_**3 **_**layer (nm)**	**Thickness of Si layer (nm)**
1	5	8
2	5	17
3	5	25
4	5	42

## Results and discussion

**Figure 1:**
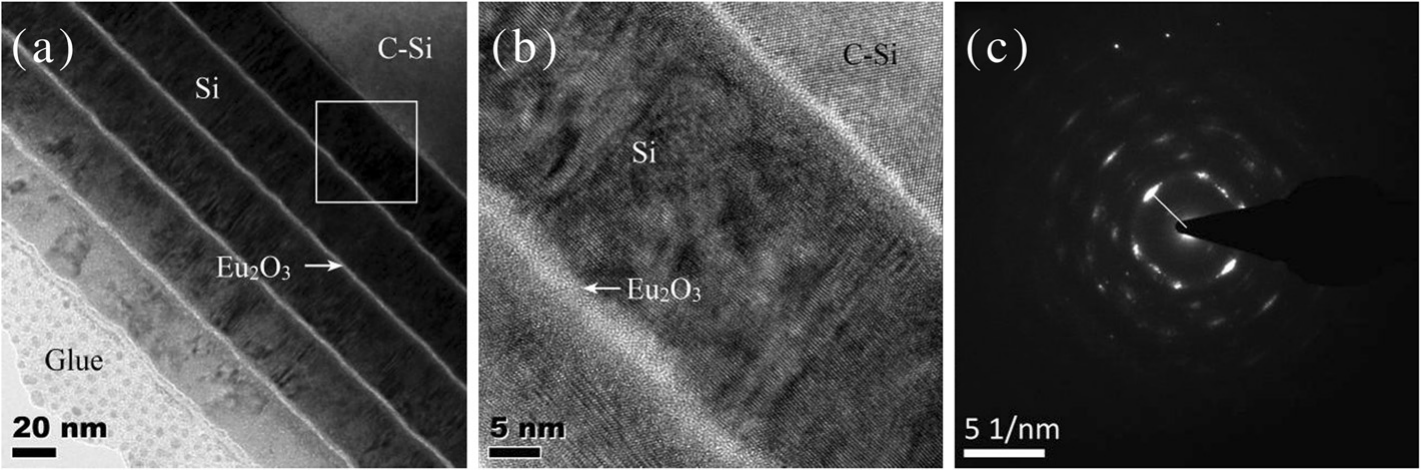
**Cross-sectional TEM images of as-deposited sample 3.** (**a**) Full view of the film, (**b**) partial enlarged view of the film, and (**c**) the SAED image of the film.

The cross-sectional TEM images of as-deposited sample are shown in Figure 1a,b. The film thickness is about 150 nm, with 5 nm in the Eu_2_O_3_ layer and 25 nm in the Si layer in one period. The interface between Eu_2_O_3_ and Si is very sharp and clear. Moreover, multicrystalline Si has formed in Si layers in the as-deposited sample, which has also been confirmed by SAED, as shown in Figure 1c. The interplanar spacing (*d*) is about 3.11 Å from the radius of the primary diffraction ring, which agrees with the *d* of the Si (111) plane. We think that the high substrate temperature and the Eu_2_O_3_ layer may induce Si crystallization.

**Figure 2:**
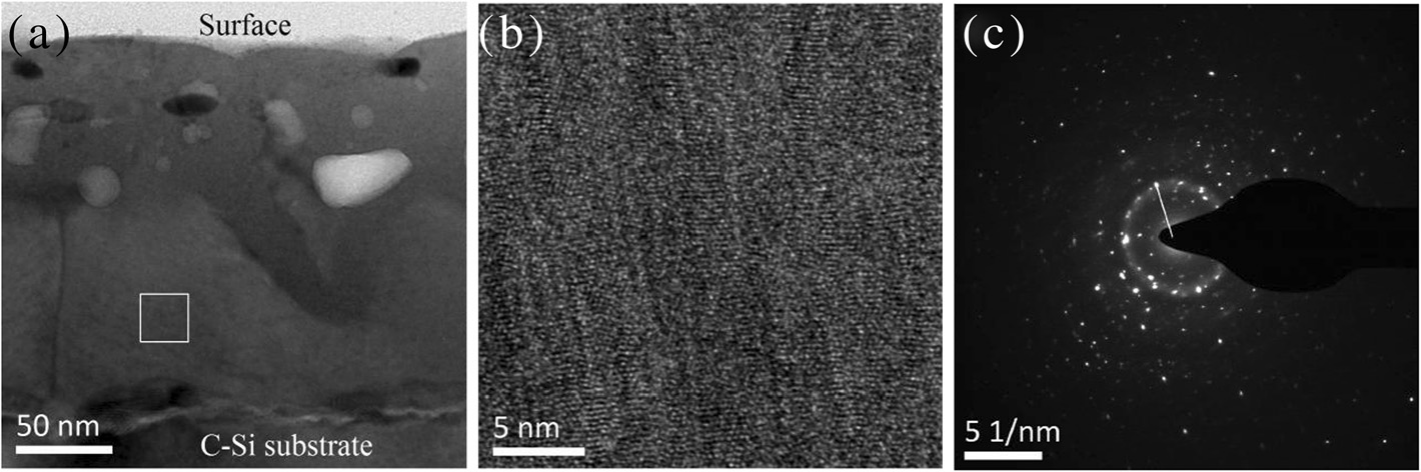
**Cross-sectional TEM images of the annealed sample 3.** (**a**) Full view of the film, (**b**) partial enlarged view of the film, and (**c**) the SAED image of the film.

**Figure 3:**
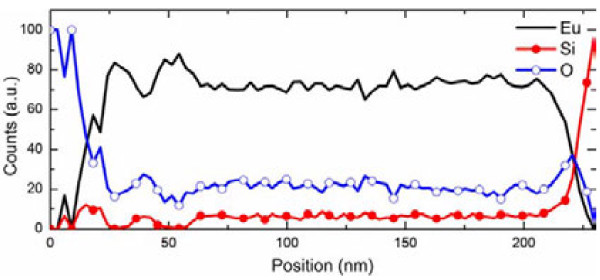
The spectra of Eu, Si, and O distribution with thickness.

Figure 2a,b shows the TEM cross section of the sample with a Si layer thickness of about 25 nm after annealing at 1,000°C for 30 s in N_2_ ambient. The interfaces between Eu_2_O_3_ layers and Si layers became blurry. This indicates that the strong reaction between Eu_2_O_3_ and Si has happened. Moreover, the crystalline europium silicate had been formed through solid-state reaction, as proven by SAED, which shows a multicrystalline ring in Figure 2c. From the SAED figures, the annealed film gave a totally different pattern compared with the as-deposited film. A lot of diffraction spots were distributed randomly, which may be ascribed to the different crystalline structures of europium silicate. In order to investigate the element distribution after the annealing process, STEM measurements were also carried out. As shown in Figure 3, Si, Eu, and O are distributed homogeneously along the thickness, suggesting that Eu_2_O_3_ and Si reacted completely in each layer.

**Figure 4:**
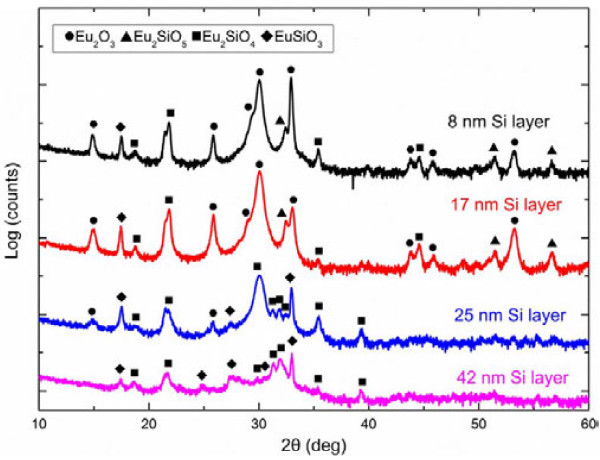
XRD patterns of the annealed samples.

The crystalline structure of the annealed films with different Si layer thicknesses was performed using XRD measurements, as shown in Figure 4. The XRD spectrum of the sample with 8-nm Si layer shows that Eu_2_O_3_, Eu_2_SiO_5_, Eu_2_SiO_4_, and EuSiO_3_ are mixed in the film after the annealing process. The corresponding JCPDS card numbers are 43-1008 (Eu_2_O_3_), 43-1009 (Eu_2_O_3_), 40-0286 (Eu_2_SiO_5_), 22-0286 (Eu_2_SiO_4_), and 35-0297 (EuSiO_3_). Eu_2_O_3_ peaks are stronger and sharper than the other peaks, suggesting that Eu_2_O_3_ is the major phase in the film due to the lack of Si. For the sample with a thicker Si layer, the XRD pattern was similar, but the Eu_2_O_3_ peak intensity had decreased. This is because more Eu^3+^ ions were involved in the reaction with increasing Si layer thickness. The sample with 25-nm Si layer exhibited different XRD patterns compared with the first two samples. The peaks corresponding to Eu_2_O_3_ and Eu_2_SiO_5_ (Eu^3+^) nearly disappeared, while the peaks corresponding to Eu_2_SiO_4_ became stronger. This indicates that Eu_2_SiO_4_ is the major phase in the film now. Moreover, through RBS measurements, the atomic concentrations of Eu, Si, and O were about 28, 14, and 58 at.% in the annealed film, which are very close to stoichiometric value of Eu_2_SiO_4_, which is consistent with the XRD results. This is interesting since the tetrahedron structure [SiO_4_]^4−^ can prevent Eu^2+^ oxidation and energy transfer among the Eu^2+^ ions by isolating the Eu^2+^ ions with [SiO_4_]^4−^. Thus, Eu^2+^ in [SiO_4_]^4−^ can exhibit longer stabilization and higher efficiency, which is already used in commercial phosphor such as Eu-doped silicate. By further increasing the Si layer thickness to 42 nm, Eu_2_O_3_ reacted with Si totally, and the Eu_2_O_3_-related peaks disappeared completely, as demonstrated by the XRD spectrum. Now, the film is mainly composed of Eu_2_SiO_4_ and EuSiO_3_ (Eu^2+^). This is consistent with Bellocchi's work where abundant Si may cause the formation of EuSiO_3_[16]. Finally, it is noticeable that most Eu ions in the sample with Si layer thicknesses between 25 and 42 nm became divalent (Eu_2_SiO_4_ and EuSiO_3_) after thermal treatments. This suggests that Eu^2+^ silicate can be achieved by precisely controlling the Eu_2_O_3_ and Si layer thicknesses.

**Figure 5:**
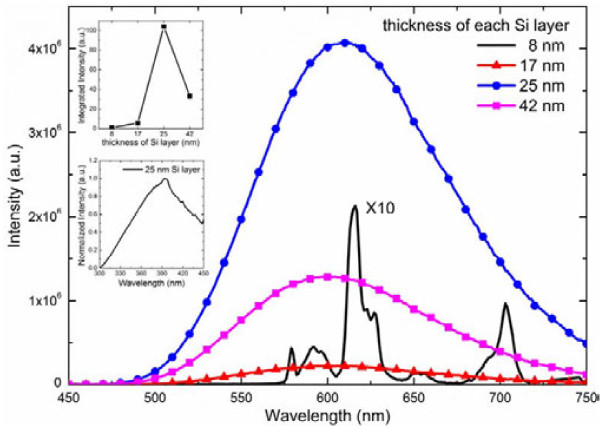
**RT PL spectra of the annealed samples.** Excitation was 365 nm, and it was obtained by HORIBA Nano Log equipped with a 450-W Xe lamp. The spectrum of sample 1 is magnified tenfold. The top left inset shows the integrated intensity of the samples. The left inset shows the PLE spectrum of annealed sample 3 monitored at 610 nm.

Figure 5 shows the RT PL spectra of the annealed samples, excited by 365-nm light. The intensity of the emission peak from sample 1 (with 8-nm Si layer thickness) was very weak. The spectrum had a sharp main peak centered at 616 nm with full width at half maximum (FWHM) of about 10 nm, corresponding to the ^5^D_0_ → ^7^F_2_ transition of Eu^3+^ ions; the other weak peaks centered at 579, 592, 653, and 703 nm, corresponding to the ^5^D_0_ → ^7^F_0_, ^5^D_0_ → ^7^F_1_, ^5^D_0_ → ^7^F_3_, and ^5^D_0_ → ^7^F_4_ transitions of Eu^3+^ ions, respectively. This indicates that most Eu ions are still trivalent in sample 1, which agrees with the XRD results. Compared to sample 1, other samples exhibited different PL spectra. They showed strong and broad band emissions, having the maximum peak at about 610 nm and FWHM at about 130 nm, which are typical dipole-allowed 4*f*^6^5*d* → 4*f*^7^ transitions of Eu^2+^ ions in Eu^2+^ silicate [16]. The red shift emission was possibly due to the fact that in Eu^2+^ silicate the Madelung potential of the negative anions around Eu^2+^ is felt less by the 5*d* electron, leading to a lowering of energy [17]. The emission peaks of Eu^3+^ disappeared in the PL spectrum of sample 2 (with 17-nm Si layer thickness ) probably because more Eu^3+^ ions in Eu_2_O_3_ layers had been deoxidized by Si, and the emission peaks of Eu^3+^ were submerged in the PL spectrum of Eu^2+^. As shown in Figure 5, the sample with 25-nm Si layer thickness has the highest PL intensity among all the samples. The integrated PL intensity of sample 3 is more than two orders higher than that of sample 1, by forming Eu_2_SiO_4_ and EuSiO_3_ through reaction with Si layer, as demonstrated in the XRD tests. However, with further increase of the Si layer thickness, the PL intensity decreased. This may be due to the formation of EuSiO_3_ crystalline structure and the residual Si.

The excitation property of sample 3 has been studied by PLE measurement from 300 to 450 nm and monitored at 610 nm. As shown in the left inset of Figure 5, the PLE spectrum exhibits a very intense and broad excitation band centered at about 395 nm, which is typical of Eu^2+^ 4*f*^6^5*d* → 4*f*^7^ transition.

Indeed, we have also grown different Si contents of Si-rich Eu_2_O_3_ films without multilayer structure. However, no Eu^2+^ ions were found after the annealing process. This indicates that divalent Eu ions only appear in the Eu_2_O_3_/Si multilayer structure. We think that in Si-rich Eu_2_O_3_ films, the Eu ions are surrounded by the Si and O ions, and Eu^3+^ silicate is formed directly during deposition. Also, it may be very difficult to form divalent Eu ions in Eu^3+^ silicate without reducing gas, even if there is abundant Si. Compared with the work of Bellocchi et al, the thickness of Si layer can be precisely controlled in nanostructure instead of the Si substrate to avoid product uncertainty. Moreover, it is reported that in silicate compounds, Eu_2_SiO_4_ is a more efficient host for Eu^2+^ light emission than the other configurations [18]. Although, in this work, the Eu trivalent state vanished in the nanostructure with increasing Si layer thickness, the divalent Eu ions exist both in Eu_2_SiO_4_ and EuSiO_3_ crystalline structures. Thus, the efficiency and intensity of Eu^2+^ light emission in Eu silicate will be further improved if the Eu_2_O_3_/Si nanostructure is optimized to prepare pure Eu_2_SiO_4_ phase.

## Conclusions

In summary, Eu silicate films were prepared by the annealing Eu_2_O_3_/Si multilayer nanostructure in N_2_ ambient. The Eu^2+^ silicates were distributed uniformly along the thickness by the reaction between Eu_2_O_3_ and Si layers. Different crystalline structures were formed and identified by changing the Si layer thickness. Through precisely controlling the thickness of Si layer in Eu_2_O_3_/Si multilayer, we have obtained Eu^2+^ silicate films, characterized by an intense and broad PL peak that centered at 610 nm. Moreover, it suggests that Eu_2_SiO_4_ phase is an efficient light emission for Eu^2+^ by forming [SiO_4_]^4−^ configuration. These results will have promising perspectives for Si-based photonic applications.

## Competing interests

The authors declare that they have no competing interests.

## Authors’ contributions

LL performed film fabrication, optical measurements, and structural measurements and also wrote the manuscript. JZ and LL analyzed the results of structural and optical characters of the samples. JZ also revised the manuscript. YZ, BC, and QW supervised the work and the text. All authors read and approved the final manuscript.
